# Brain–Computer Interface-Based Adaptive Automation to Prevent Out-Of-The-Loop Phenomenon in Air Traffic Controllers Dealing With Highly Automated Systems

**DOI:** 10.3389/fnhum.2019.00296

**Published:** 2019-09-06

**Authors:** Gianluca Di Flumeri, Francesca De Crescenzio, Bruno Berberian, Oliver Ohneiser, Jan Kramer, Pietro Aricò, Gianluca Borghini, Fabio Babiloni, Sara Bagassi, Sergio Piastra

**Affiliations:** ^1^BrainSigns srl, Rome, Italy; ^2^IRCCS Fondazione Santa Lucia, Neuroelectrical Imaging and BCI Lab, Rome, Italy; ^3^Department of Molecular Medicine, University of Rome “Sapienza,” Rome, Italy; ^4^Department of Industrial Engineering, University of Bologna, Bologna, Italy; ^5^French Aerospace Lab, ONERA, Salon Air, France; ^6^German Aerospace Center (DLR), Braunschweig, Germany; ^7^College of Computer Science and Technology, Hangzhou Dianzi University, Hangzhou, China

**Keywords:** electroencephalography, eye-tracking, vigilance, Out-Of-The-Loop, passive brain–computer interface, adaptive automation, air traffic control, human–machine interface

## Abstract

Increasing the level of automation in air traffic management is seen as a measure to increase the performance of the service to satisfy the predicted future demand. This is expected to result in new roles for the human operator: he will mainly monitor highly automated systems and seldom intervene. Therefore, air traffic controllers (ATCos) would often work in a supervisory or control mode rather than in a direct operating mode. However, it has been demonstrated how human operators in such a role are affected by human performance issues, known as Out-Of-The-Loop (OOTL) phenomenon, consisting in lack of attention, loss of situational awareness and de-skilling. A countermeasure to this phenomenon has been identified in the adaptive automation (AA), i.e., a system able to allocate the operative tasks to the machine or to the operator depending on their needs. In this context, psychophysiological measures have been highlighted as powerful tool to provide a reliable, unobtrusive and real-time assessment of the ATCo’s mental state to be used as control logic for AA-based systems. In this paper, it is presented the so-called “Vigilance and Attention Controller”, a system based on electroencephalography (EEG) and eye-tracking (ET) techniques, aimed to assess in real time the vigilance level of an ATCo dealing with a highly automated human–machine interface and to use this measure to adapt the level of automation of the interface itself. The system has been tested on 14 professional ATCos performing two highly realistic scenarios, one with the system disabled and one with the system enabled. The results confirmed that (i) long high automated tasks induce vigilance decreasing and OOTL-related phenomena; (ii) EEG measures are sensitive to these kinds of mental impairments; and (iii) AA was able to counteract this negative effect by keeping the ATCo more involved within the operative task. The results were confirmed by EEG and ET measures as well as by performance and subjective ones, providing a clear example of potential applications and related benefits of AA.

## Introduction

Over the last decade, the global air traffic growth has exhibited a fairly stable positive trend, despite economic immobility, financial crisis, and increased security concerns. According to the most recent annual global statistics provided by the International Civil Aviation Organization (ICAO, n.d.), the total number of passengers carried grew to 4.1 billion in 2017, 7.2% higher than the previous year, while the number of departures reached 36.7 million in 2017, a 3.1% increase compared to 2016. According to the latest ICAO long-term air traffic forecasts, the 4.1 billion airline passengers carried in 2017 are expected to grow to about 10.0 billion by 2040, and the number of departures is projected to rise to some 90 million in 2040. In addition, there is a concurrent incredible growth of the emerging field of unmanned aerial vehicles (UAVs), which, in the next future, are expected to become a key factor for logistics and freight transportation applications. Recently, Research and Markets (Research and Markets ltd., n.d.) estimated that the overall UAV market was valued at $18.14 billion in 2017 and projected to reach $52.3 billion by 2025, at a compound annual growth rate of 14.15%.

Therefore, it is clear that air traffic flow patterns will become more complex, making situations and conflicts harder to identify for a human operator, putting immense pressure on the air traffic control system (Hopkin, 2017). In this context, several solutions have been proposed for modernizing air traffic control and meet the demands for enhanced capacity, efficiency, and safety. All of them had the same common denominator: the automation (Hilburn et al., 1998).

Over the past 50 years, automation technology has actually changed our modern society. Perhaps there is no facet of everyday life in which the influence of automation technology has not been felt.

Whether at work or at home, while traveling or while engaged in leisurely pursuits, human beings are becoming increasingly accustomed to using and interacting with sophisticated systems designed to assist them in their activities. Most safety-critical systems—power plants, intensive care units, and so on—already include automation. Even more radical changes are expected in the future with increase in computer performance. The explosive growth of microprocessor technology (rapid improvements in computer performance, together with a decrease in size, cost, and power consumption) makes automation of many systems a reasonable alternative to traditional manual operation, sophisticated automation is becoming ubiquitous, and air traffic management will not appear as an exception (Borghini et al., 2017a). Crucially, whatever the advantages of using any particular automation technology, it is clear that it has profoundly changed human activity. In fact, automation is defined as the process of entirely or partially allocating the activities constituting a task usually performed by a human, to a machine or a system (Parsons, 1985). In such definition, automation refers to the full or partial substitution of a function initially performed by the human operator. In that sense, automation is not all or none but can vary across a continuum of levels, from the fully manual performance to the full automation. To this regard, different scales of levels of automation (LOAs) involving automation of decision-making and action have been proposed (see, for example, Sheridan, 1992; Zellweger and Donohue, 2001; Sheridan, 2002). Further, automation also includes information gathering and analysis. For example, air traffic controllers’ (ATCos’) tasks include (a) the acquisition of radar information on location, flight plans and identity of many aircraft, weather information, and so on; (b) the combination and analysis of the appropriate information; (c) decision-making (the speed, heading, and altitude that different aircraft must maintain for a safe separation and to bring the aircraft safely through a sector of airspace or to land or take off) regarding the situation decisions to be made; and finally (d) a means to get the pilots (and aircraft) to cooperate and execute the instructions given. As a result, Parasuraman et al. (2000) have suggested an extension of the LOA concept to four information-processing stages: (a) information acquisition, (b) information analysis, (c) decision-making, and (d) action, with each stage having its own LOA scale (for similar scales, see Endsley and Kaber, 1999).

Automation in the aeronautical field has been recognized as an important topic (Amaldi and Quercioli, 2016); however, the interposition of automated systems between ATCos and processes will dramatically transform the nature of their work (Wickens et al., 1998). Understanding the characteristics and the dynamics of this transformation is vital for successful design of new automated systems. When a new automation solution is introduced into a system, or when there is an increase in the autonomy of automated systems, developers often assume that adding “automation” is a simple substitution of a machine activity for human activity (the so-called “substitution myth”; Sarter et al., 1997). However, the fascination regarding the possibilities afforded by technology often obscures the fact that automation also produced new loads and difficulties for the humans responsible for operating, troubleshooting, and managing high-consequence systems. In such system, the main role for humans will be to undertake what is called supervisory control (Sheridan, 2002). In other words, it is expected that ATCos will be relegated to the role of monitoring and decision-making, keeping an eye on deviations and failures, and taking over when necessary. This new form of interaction will differ dramatically from the traditional interaction of the ATCos with tools and devices that possess no intelligence, in which all sensing and control were done by the human operator. The key difference between passive information processing (future highly automated scenario) and direct action on the process (current scenario) is that the former involves functions similar to those maintained during process monitoring (e.g., scanning information sources), whereas the latter involves manual control functions including process planning, decision-making, selecting responses, and implementing strategies.

Therefore, if, on the one hand, implementing higher LOAs can improve the efficiency and capacity of the ATM service, on the other hand, it can also have negative effects on the performance of human operators (Parasuraman et al., 2000; Langan-Fox et al., 2009; Aricò et al., 2017b). For example, it can reduce the vigilance and sensitivity to important signals (Billings, 1991); it can create unjustified, excessive trust and complacency in system ability (Parasuraman et al., 1993); and it can lead to a loss of operator situation awareness, because of the reduced or even excluded interaction among ATCos and aircraft pilots (Endsley and Kiris, 1995), and loss of cognitive and manual skills (Parasuraman, 2000). These effects have been observed in all those domains in which the LOA is already increased, apart from aviation (Endsley, 1999; Parasuraman et al., 2008), such as nuclear power plants (Norman, 1988), and the stock market (Jones, 2013). Indeed, it is now well accepted that automation can have negative consequences for performance and safety due to these difficulties (Endsley and Kaber, 1999; Parasuraman et al., 2000). This set of difficulties related to the poor human performance as “automation supervisor” is called the Out-Of-The-Loop (OOTL) phenomenon (Kaber and Endsley, 1997; Jones et al., 2009). In other words, the OOTL phenomenon corresponds to a lack of control loop involvement of the human operator. Automation technology is expected to create an increasing distance between ATCos and the loop of control, making him disconnected from the automation system. Such a removal could lead to a decreased ability of the ATCos to intervene in system control loops and assume manual control when needed in overseeing automated systems (Endsley and Kiris, 1995; Merat and Jamson, 2009). In the current context of a continued increase in automation, understanding the sources of difficulties in the interaction with automation and finding solutions to compensate such difficulties are crucial issues for both system designer and human factor society. Detecting the occurrence of this phenomenon, or even better detecting the dynamics toward this degraded state, is an important issue in order to develop tools for operators’ evaluation and monitoring and hopefully human error prevention.

A holistic approach is to develop automation in such a way that it can be seen as a partner. Human operator and automation should form a team that works cooperatively together, in a highly adaptive way to achieve its objectives (Klien et al., 2004). They have to adapt to each other and to the context in order to guarantee fluent and cooperative task achievement (Christoffersen and Woods, 2002). A technical solution for some of these challenges is the concept of adaptive automation (AA) (Scerbo, 1996; Kaber and Endsley, 2004); i.e., the system is able to adapt its behavior to the needs and the state of the user in real time. It is able to meet the changing needs of operators often without requiring the human operator to explicitly state his needs or trigger the adaptations. The concept of AA relies on the dynamic allocation of function between operators and systems. This means that the LOA of such system is not fixed but is adapted during the runtime according to the current needs of the operator (Inagaki, 2003). With respect to the OOTL issue, AA would be able to counteract, keeping the operator *in the loop* by dynamically assigning him/her manual actions (Kaber and Endsley, 1997).

However, a critical challenge remains: what should determine and “trigger” on-time allocation of functions between the operator and the automation system. Three main invocation techniques have been proposed (Parasuraman et al., 1992): (i) logic based on specific events that occur in the task environment; (ii) estimation of operator behavioral performance in real time and use deviations from acceptable ranges to invoke the automation; and (iii) psychophysiological measures able to assess in real time the ATCo’s mental state and to use it to trigger changes among the modes of automation (Byrne and Parasuraman, 1996). The latter are receiving increasing attention from the scientific community because of some intrinsic advantages related to their application, also thanks to the great improvements produced by the research in passive brain–computer interfaces (BCIs) (Zander and Kothe, 2011; Aricò et al., 2017c, 2018; Antonacci et al., 2017). First, the measures can be obtained continuously with little intrusion, i.e., without interrupting the operator’s work with additional tasks or questions (Aricò et al., 2016b). Second, it is difficult to measure resource capacity with performance indices because behavior is often at a low level when humans interact with automated systems. Also, any eventual performance degradation would become “measurable” by the system when the operator already suffered a mental impairment, i.e., “after the fact” (Endsley, 1995). Finally, these measures have been found to be reliably diagnostic of multiple levels of arousal, attention, and workload (Berka et al., 2004; Giraudet et al., 2015; McMahan et al., 2015; Ahlstrom et al., 2016; Dehais et al., 2016; Borghini et al., 2017b, c; Cartocci et al., 2018; Dehais et al., 2018; Di Flumeri et al., 2018). Even if there are still many critical conceptual and technical issues (e.g., making the recording equipment less obtrusive and more comfortable and obtaining reliable signals in noisy environments) (Minguillon et al., 2017; Aricò et al., 2018), numerous works have proved that it is indeed possible to obtain indices of user’s brain activity and use that information to drive an AA system to improve performance and moderate workload in complex environment (see, for example, Wilson and Russell, 2003; John et al., 2004; Aricò et al., 2016a). Therefore, as previously introduced, such a kind of application, i.e., to covertly evaluate the user’s mental state and to use this information as a mono-directional communication channel toward a machine/computer, is generally named passive BCI (Zander and Kothe, 2011; Aricò et al., 2017c, 2018). To this regard, several neuroimaging techniques have been shown to provide reliable evidences of changes in vigilance, suggesting them as potential candidates for AA such as electroencephalography (EEG), near-infrared spectroscopy (NIRS), and functional magnetic resonance imaging (fMRI), as well as brain-unrelated techniques such as electrocardiogram (ECG) or skin electric potential (GSR).

Among these techniques, EEG is regarded as the “gold standard” of vigilance detection, especially if regarding applications outside the laboratory, i.e., in real contexts (Oken et al., 2006; Aricò et al., 2017c; De Crescenzio et al., 2017). Nowadays, there is a very large literature concerning the relationship of oscillatory activity and attention/vigilance, and brain dynamics associated to vigilance are well known (Frey et al., 2015). Overall, there is increased slow frequency activity (alpha and theta bands) on the EEG with decreasing vigilance, whereas increasing vigilance induces an increase in beta activity (Borghini et al., 2014). For example, Lin et al. (2005) explored many experimental results to verify the relationship between EEG power spectrum density (PSD) and drowsiness. They observed that the power of alpha and beta rhythm in an alert state was greater than in a drowsy state (see also; O’Connell et al., 2009; Martel et al., 2014). Brookhuis and de Waard (2010) described how, in driving simulator research, analysis of EEG by means of power density spectra might indicate driver vigilance state, with particular interest in drowsiness and loss of sleep. Beta activity (12–30 Hz) is predominant when the participant in the study is generally awake and alert, while the activity dropping to alpha activity (8–12 Hz) indicates developing drowsiness, and going further down into the theta region (5–8 Hz) may even lead to falling asleep. Recently, several investigators have reported that EEG power band ratios may be better at distinguishing among different levels of attention than is any single power band. Pope et al. (1995) described a system in which changes between modes of automation were triggered by an index of engagement (EI) based on ratios of EEG power bands (alpha, beta, theta, etc.). The rationale for the EI is that increases in arousal and attention are reflected in the beta-bandwidth while decreases are reflected in the alpha and theta bandwidths. They studied several different engagement indices from a variety of sites. The engagement indices were computed using a 40-s moving window that was updated every 2 s. Such result was replicated and extended by several works (Mikulka et al., 2002). Taken together, these different works showed that continuous, accurate, non-invasive, and nearly real-time estimation of vigilance levels using EEG power spectrum analysis is feasible.

In this context, the present work aims at describing the conception and the validation of the so-called “Vigilance and Attention Controller” (VAC), a system based on EEG and eye-tracking techniques. The VAC’s main function is to assess in real time the vigilance level of an ATCo dealing with a highly automated interface and to use this measure to adapt the LOA of the interface itself, therefore employing BCI technology. Such system has been developed within the framework of the Sesar Joint Undertaking H2020 European Project “MINIMA” (Minima Project, n.d.). The system has been tested at the University of Bologna on 14 professional ATCos dealing with a real ATM interface developed by the Deutsches Zentrum für Luft- und Raumfahrt e.V. (DLR, i.e., the German Aerospace Center). The interface has been designed according to what is expected in the next decades, i.e., with the highest LOA. However, the interface automation level can be lowered through proper external triggers, in this case provided by the VAC. The ATCos performed two high realistic scenarios, one with the VAC disabled (i.e., the automation level was kept fixed at the maximum level during the whole scenario) and one with the VAC system enabled (i.e., AA). During the experiment, performance and subjective measures have also been collected, in order to provide an overview of the problem from different perspectives, as well as a comprehensive evaluation of the VAC system.

## Materials and Methods

### The Experimental Design

Fourteen voluntary subjects, all males, professional ATCos from Ente Nazionale per l’Assistenza al Volo (ENAV, i.e., the Italian air navigation service provider), participated in the study (average age of 45.0 years, *SD* = 7.5). The experiment took place at the Virtual Reality Lab of the University of Bologna in Forlì (Italy). All participants were naive to the purposes of the study. The experiment was conducted following the principles outlined in the Declaration of Helsinki of 1975, as revised in 2000. Informed consent and authorization to use the video graphical material were obtained from each subject through a written and signed form, after the explanation of the study. However, participants have been informed about the study’s purpose only after the experiment.

**FIGURE 1:**
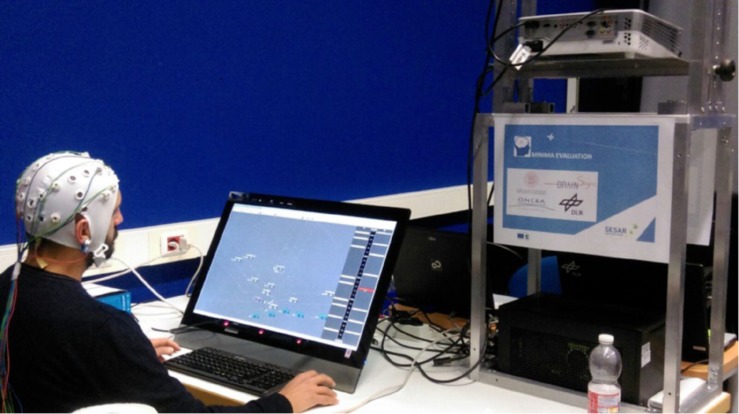
The figure shows a moment of the experimental task. The ATCo is supervising a traffic situation over the ATM interface.

Participants were seated in a comfortable armchair with an appropriate height in front of the air traffic control (ATC) simulator, installed on a 27-inch computer screen (Berberian et al., 2017). The distance from the screen to the plane of the subject’s eyes was 60 cm. They had to perform an ATC task. A highly automated terminal maneuvering area (TMA) had been selected as use case (see Figure 1). The subject was instructed to monitor arriving and departing traffic and to intervene only in cases of conflicts or emergencies. The next paragraphs will describe in detail such experimental platform.

#### The ATC Simulator

All the traffic scenarios were supported by an ATC simulator that was responsible for proceeding radar tracks of each aircraft. This simulator also provided the aircraft behavior triggered by automatically executed controller commands in all simulations. Those controller commands (e.g., DESCEND, REDUCE) were calculated by an arrival manager (AMAN; Helmke et al., 2009) and sent to the simulator on time. Nevertheless, the controller was still able to insert additional commands for each aircraft *via* the mouse interface of the radar display. Departure aircraft radar tracks were also generated by an air traffic simulator without following AMAN trajectory calculation and automatic commands. All scenarios ensured that they are almost free of conflicts except of those conflicts the controller should detect in very seldom cases (Ohneiser et al., 2018).

##### The AMAN

All trajectory planning was done by a software-based AMAN. The AMAN software consists of several modules: A lateral path predictor, an arrival interval calculator, and a scheduler. In combination, these modules are capable of calculating arrival sequences for aircraft within a specified TMA. Aircraft movement was processed through a dedicated air traffic simulator for flight movements.

##### Radar display: radarvision

**FIGURE 2:**
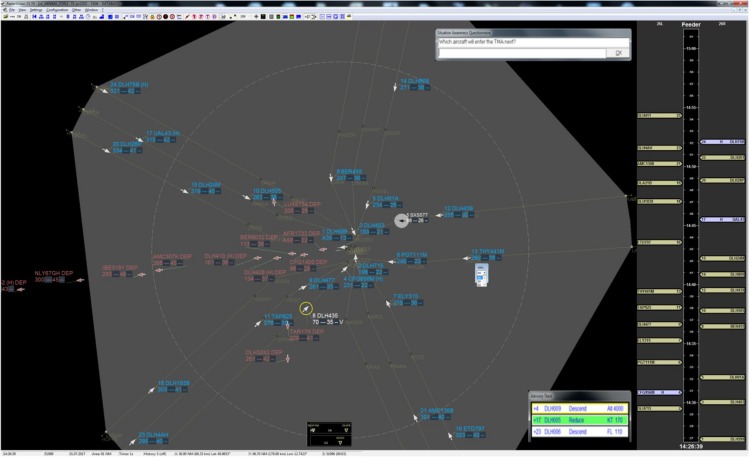
The figure shows a screenshot of the ATM interface (RadarVision). The central view consisted of the situation data display (SDD) that displays runways, TMA borders, routes, points, and aircraft. The timeline was shown right of the SDD. Each aircraft had a label dedicated to a certain time and runway.

Visualization of radar data calculated within the simulation software was done *via* the RadarVision display. RadarVision visualizes static airspace dependent data as well as calculation results from the AMAN. The central view consisted of the Situation Data Display (SDD) that displays runways, TMA borders, routes, points, and aircraft. By using the “mouse over” functionality on an aircraft icon, corresponding data like the planned 4D trajectory or weight category could be visualized in an extended label. A timeline was shown right of the SDD. Each aircraft had a label dedicated to a certain time and runway. All dynamic elements moved downwards as time went on (see Figure 2).

RadarVision also served as a human–machine interface as it allowed the controller to give clearances to the aircraft displayed within the TMA. In RadarVision, mouse control interfaces were used to give commands to the aircraft within the controller’s area of responsibility.

##### Automation solutions

Two different LOAs were implemented within the ATC Simulator: Level 2, characterized by the highest level of automated tasks (i.e., the operative situation expected during the next decades), and Level 0, during which the LOA is reduced and some tasks have to be manually executed by the operator. The concept beyond this design was that Level 2 is suitable while the ATCo’s vigilance is appropriate, instead in case of vigilance decreasing the automation level should be reduced to Level 0 in order to enhance their task engagement. Table 1 provides an overview of the main features distinguishing the two automation levels.

**TABLE 1:** The table summarizes the main features distinguishing the two automation levels (2 and 0) implemented within the ATC simulator.

**ATC task**	**Level 2 (High automation)**	**Level 0 (Low automation)**
*Air-ground communication (Datalink)*	Automatic assumption. Manual speed and altitude clearances.	Manual assumption, speed and altitude clearances.
*Attention guidance (Eye-Tracker)*	Disabled.	The aircraft was highlighted on the screen if not looked for more than the time the aircraft itself takes to cover a distance of 1.5 Nautical Miles.
*Attention guidance if short-term conflicts predicted*	Disabled. Situation automatically solved.	If the system predicted a potential conflict within 60 s, related aircrafts were highlighted. Manual action requested.
*Attention guidance if trajectory deviations or loss of separation*	Disabled. Situation automatically solved.	If the system detected unforeseen trajectory deviations or loss of separation, related aircraft was highlighted. Manual action requested.
*Attention guidance if unsatisfied scheduling*	Disabled. Situation automatically solved.	If the system detected an aircraft unsatisfying the scheduled times, related aircraft was highlighted. Manual action requested.
*Centerline Separation Range (CSR) system – support interface displaying landing separation times*	Hidden.	Visible.
*Advisories/Messages*	Do not require any manual action.	ATCo’s manual actions requested.
*Sector size*	Standard.	Increased to stimulate ATCo’s engagement.
*Situation Awareness questions*	Disabled.	Questions about the current traffic situations were displayed on the screen to stimulate ATCo’s engagement.

#### The Tasks

Three different scenarios were designed: a training one (hereinafter called “TRAINING”) and two experimental ones (hereinafter called “BASELINE” and “SOLUTION”). In each of them, approach and departure air traffic inside the TMA were simulated. Each scenario lasted 45 min. In particular, real traffic data of the International Munich Airport (two runways) have been used. Traffic conditions and flow were different but comparable. There was a rate of roughly 30 arrivals per hour and runway as well as 15 departures per hour and runway. Scenario did not contain overflights. In total, about 100 aircraft were present in each scenario. The weight category of aircraft consisted of 10% “Heavy” and 90% “Medium.” Therefore, airspace was quite dense since in future scenarios automation is expected to optimize traffic flow during normal operations. Typical call signs of Munich airport were used but changed between different scenarios to avoid learning effects (Borghini et al., 2017c). The starting points of aircraft were outside the TMA. These points were moved to different positions almost semi-circle-wise rotating around the runways due to airspace structure (again to avoid learning effects of participants). Few conflicts were introduced in each scenario as it was considered a well-functioning automation with only very few necessary controller actions. Accordingly, participants were instructed to actively intervene only in case of danger of separation losses between aircraft.

Additionally, a short scenario (15 min, hereinafter called “CALIBRATION” scenario) was designed very similar to the BASELINE one, with the automation thus fixed on Level 2. It was to calibrate the VAC on each operator before starting the experiment (please refer to the section *Electroencephalographic Signal Recording and Processing*).

##### TRAINING scenario

The TRAINING scenario was used to introduce the study participants, i.e., the Controllers, to the MINIMA concept. It aimed (1) to familiarize participants with the VAC, in order to avoid any possible confounding behavior due to the learning effect (Borghini et al., 2017c), and (2) to cause subjects to trust the system and therefore increase their willing of using it during their work. During the TRAINING scenario, the automation level was manually altered by the experimenters. This served to provide subjects with a standardized introduction to the task environment in both low and high automation modalities. TRAINING consisted of 15 min of high-level automation, 15 min of low-level automation, and finally another 15 min of high-level automation.

##### BASELINE scenario

The BASELINE scenario served as a reference scenario for how air traffic management will be done in the next decades. While most of the work is left to a highly automated system, the human operator’s role was reduced to that of a mere supervisor during this scenario. Therefore, the LOA was set on Level 2 (please refer to Table 1) and kept fix along the whole scenario.

##### SOLUTION scenario

In the SOLUTION scenario, the integrated VAC developed for MINIMA actively adapted the LOA within the task environment, based on the operators’ vigilance online measured *via* EEG data. In particular, the proper LOA was automatically set and eventually switched from Level 2 to 0 (please refer to Table 1), and vice versa, every 5 min on the basis of the overall EEG-based vigilance scores. When controllers showed mainly low levels of vigilance during the last time frame, the LOA was lowered (Level 0). On the contrary, if controllers showed high levels of vigilance, automation was set back to the highest level (Level 2). Depending on the LOA, controllers were either reallocated part of their operative tasks, i.e., manually manage traffic, or were provided with additional information such as unmonitored aircraft and potential separation losses (please refer to Table 1).

##### CALIBRATION scenario

The aim of the CALIBRATION scenario, 15 min long, was to provide to the VAC preliminary calibration EEG data of the specific operator who is going to perform the experiment. In other words, the VAC algorithms based on machine learning employed these data to develop the subjective vigilance classification model, as well as the threshold discriminating high and low vigilance states (please refer to the section *Electroencephalographic Signal Recording and Processing*).

Therefore, this calibration task had to be designed in order to induce both high and low vigilance states. For this reason, the automation level was kept fixed on Level 2 (the highest one). Essentially, as for the BASELINE scenario, the controller had just to monitor the traffic. From a scientific point of view, long monotonous tasks used to induce vigilance decreasing is demonstrated (Thackray et al., 1974), with significant effects after 10 min (Loh et al., 2004).

In addition, in order to enhance the operators’ vigilance at the beginning of the task and induce a consecutive relaxation, two standardized questions about the current traffic situation were asked during the first 5 min. Actually, before the beginning of this calibration phase, the subject was informed of the occurrence of two questions. However, he was told these questions could be provided along the whole 15 min, in order to avoid any expectation and to induce relaxation after the second question. In particular, the two questions were:

(1) At minute 3, “How many aircraft are under your control?”
(2) At minute 5, “Which is the altitude of the lower aircraft that is approaching the runway?”

#### The Experimental Protocol

The experimental protocol was developed along two sessions when applicable in two consecutive days, in order to avoid possible interfering factors such as fatigue and drowsiness (Pattyn et al., 2008; Borghini et al., 2014; Körber et al., 2015).

In the first session, subjects were introduced with the experimental task and the automation systems, and they performed the TRAINING scenario. No behavioral and physiological data were collected during this session.

The second session was the actual experimental session during which operators’ performance with and without the AA solutions was compared. They performed once either the BASELINE or the SOLUTION scenario in a randomized way, in order to avoid any bias in the results due to the order of task execution (Urbach, 1985).

This experimental session started with a briefing to clarify any eventual operator’s doubt or still open question. Afterward, the EEG and eye-tracking systems were installed and calibrated (please refer to the section *The Data Collection*). At this point, the controller was asked to perform the CALIBRATION scenario (please refer to the section *CALIBRATION Scenario*) in order to calibrate the VAC on his own brain activity features, i.e., to calculate individual parameters as well as the threshold for discriminating low and high vigilance states. Then, the subjects completed the BASELINE and SOLUTION scenario. EEG and eye-tracking data were gathered during both scenarios, but they were actively used online only during the SOLUTION one. After each scenario, subjects completed two electronic questionnaires. The first questionnaire was an adapted version of the Dundee Stress State Questionnaire (DSSQ; Cavalcanti and Azevedo, 2013). The second questionnaire was the NASA Task Load Index (NASA-TLX; Hart and Staveland, 1988).

The experimental session was closed by a debriefing, during which subjects were told about the experiment’s actual purpose.

**FIGURE 3:**
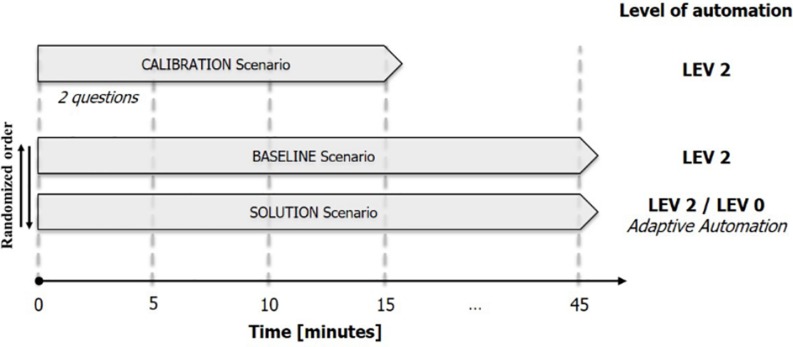
Graphical summary of the experimental task: after the short CALIBRATION scenario, the ATCos performed once either the BASELINE and SOLUTION scenario. These two scenarios were performed in a randomized order among subjects to avoid any bias.

In Figure 3, a graphical summary of the experimental tasks is reported.

#### The Data Collection

##### Electroencephalographic signal recording and processing

EEG data have been recorded through the g.USBamp (Guger Technologies GmbH, Austria), a wired EEG system. In this case, the sampling frequency was set on 256 Hz. Fifteen traditional Ag/AgCl electrodes were placed mainly on the prefrontal, frontal, and centro-parietal sites, according to scientific literature (Makeig and Inlow, 1993; Berka et al., 2007; Borghini et al., 2014) and preliminary validation experiments performed during MINIMA project (Minima Website, n.d. please refer to References section). In particular, the EEG setup included Fpz (it has been used only for ocular artifacts rejection), AF3, AF4, AF7 AF8, Fz, F3, F4, F7, F8, CP3, CP4, Pz, P3, and P4, according to the 10–20 International System. A pair of electrodes on the earlobes has been used as reference, while the system ground has been placed on the left mastoid. The impedance of all electrodes was kept below 20 kΩ.

The recorded EEG signals were then entirely digitally processed online. In particular, the signals were band-pass filtered (1–30 Hz, 5th-order Butterworth filter) and the Fpz channel has been used to remove eye-blink artifacts from the EEG data by using the regression-based algorithm REBLINCA (Di Flumeri et al., 2016). With respect to other regressive algorithms (e.g., Gratton et al., 1983), the REBLINCA algorithm has the advantage of preserving EEG information in blink-free signal segments by using a specific threshold criterion that automatically recognizes the occurrence of an eye-blink, and only in this case does the method correct the EEG signals. If there is no blink, the method has no effect on the EEG signal. In addition, the REBLINCA method does not require EOG signal(s), thus reducing system invasiveness and increasing subject comfort. Then, the EEG signal has been segmented into epochs of 2 s, shifted of 0.125 s. This windowing has been chosen with the compromise of having both a high number of observations, in comparison with the number of variables, and to respect the condition of stationarity of the EEG signal (Elul, 1969). In fact, this is a necessary assumption in order to proceed with the spectral analysis of the signal. At this point, specific procedures of the EEGLAB toolbox (Delorme and Makeig, 2004) have been employed to remove any other kind of artifacts, such as environmental noise, controllers’ movements, etc., that are generally avoided in laboratory but become largely impacting in highly realistic conditions as those of the present study (Minguillon et al., 2017; Di Flumeri et al., 2018). Specifically, the EEG epochs with the signal amplitude exceeding ±100 μV (*Threshold criterion*) were marked as “artifact.” Then, each EEG epoch has been interpolated in order to check eventual abnormal drifts of the signal within the considered epoch (*Trend estimation*). To detect such drifts, a linear trend fitting the EEG data with *R*^2^ > 0.3 is computed. If the slope of the linear trend was higher than 10 μV/s, the considered epoch was marked as “artifact.” Finally, the signal sample-to-sample difference (*Sample-to-sample criterion*) has been analyzed: if such a difference, in terms of absolute amplitude, was higher than 25 μV, i.e., an abrupt variation (non-physiological) happened, the EEG epoch is marked as “artifact.” At the end, the EEG epochs marked as “artifact” have been removed from the EEG dataset with the aim to have a clean EEG signal to perform the analyses.

Once the EEG dataset is cleaned, the power spectral density (PSD) was calculated for each EEG channel for each epoch using a Hanning window of the same length as the considered epoch (2 s long, which means 0.5 Hz of frequency resolution). Then, the EEG frequency bands of interest have been defined for each subject by the estimation of the *individual alpha frequency* (IAF) value (Klimesch, 1999). In fact, it has been demonstrated that brain rhythms, generally assumed with fixed frequency bands (e.g., theta is the band between 4 and 8 Hz), suffer slight shifting over the frequency domain because of age, diseases, and even more across different subjects (Doppelmayr et al., 1998). Therefore, a more precise definition of brain rhythms in bands taking into account eventual individual differences is possible by defining all the bands as a function of the IAF, i.e., the peak of the power spectrum within the traditional alpha range (Klimesch, 1999). In order to have a precise estimation of the alpha peak and, hence, of the IAF, the subjects were asked to keep their eyes closed for a minute before starting the experimental tasks. Finally, a spectral features matrix (EEG channels × Frequency bins) has been obtained in the frequency bands directly correlated to the vigilance. In particular:

• Theta [IAF – 6 ÷ IAF – 2] and beta [IAF + 2 ÷ IAF + 16] bands over the EEG frontal channels,
• Alpha [IAF – 2 ÷ IAF + 2] band over the EEG centro-parietal channels, and
• Theta band itself over the EEG parietal channels.

were considered as variables for the online vigilance evaluation (Pfurtscheller et al., 1996; De Gennaro et al., 2005; Berka et al., 2007; Olbrich et al., 2009; Klimesch, 2012; Borghini et al., 2017a).

At this point, the automatic-stop-StepWise Linear Discriminant Analysis (asSWLDA), a specific Machine-Learning algorithm (basically an upgrade version of the well-known StepWise Linear Discriminant Analysis) previously developed (Aricò et al., 2016b), patented (Aricò et al., 2017a), and applied in different applications (Di Flumeri et al., 2015; Aricò et al., 2016a; Borghini et al., 2017b, c; Di Flumeri et al., 2018) by the authors has been employed. On the basis of the calibration dataset (CALIBRATION scenario), the asSWLDA is able to find the most relevant spectral features to discriminate the Vigilance levels of the Controllers along the experimental tasks (i.e., BASELINE and SOLUTION). In particular, the first 5 min of the CALIBRATION scenario were assumed at High Vigilance level, while the last 5 min were assumed at Low Vigilance level. Once such spectral features are identified, the asSWLDA assigns to each feature specific weights (*w_*i train*_*), plus a bias (*b*_*train*_), such that an eventual discriminant function (i.e., the model) computed on the training dataset [*y*_*train*_(*t*)] would take the value *1* in the High Vigilance condition while *0* in the Low Vigilance one. This step represents the calibration, or *Training phase* of the classifier. Later on, the weights and the bias determined during the training phase are used to calculate the Linear Discriminant function [*y*_*test*_(*t*)] during the online application, which should be between 0 (if the condition is Low Vigilance) and 1 (if the condition is High Vigilance). Finally, a moving average of 30 s (30MA) is applied to the *y*_*test*_(*t*) function in order to smooth it out by reducing the variance of the measure: its output is defined as the *EEG-based Vigilance index* (*V*_*SCORE*_).

Here, below the training asSWLDA discriminant function [Equation 1, where *f*_*i train*_(*t*) represents the PSD matrix of the training dataset for the data window of the time sample *t*, and of the *i*th feature], the testing one [Equation 2, where *f*_*i test*_(*t*) is as *f*_*i train*_(*t*) but related to the testing dataset] and the equation of the *EEG-based Vigilance index* computed with a time resolution of 30 s (*V*_*SCORE*_, Equation 3) are reported.

(1) $$y_{t\,r\,a\,i\,n}\,\left(t\right)=\sum_{i}w_{i\,t\,r\,a\,i\,n}\cdot f_{i\,t\,r\,a\,i\,n}\,\left(t\right)+b_{t\,r\,a\,i\,n}$$

(2) $$y_{t\,e\,s\,t}\,\left(t\right)=\sum_{i}w_{i\,t\,r\,a\,i\,n}\cdot f_{i\,t\,e\,s\,t}\,\left(t\right)+b_{t\,r\,a\,i\,n}$$

(3) $$V_{S\,C\,O\,R\,E}=30\,M\,A\,\left(y_{t\,e\,s\,t}\,\left(t\right)\right)$$

#### Eye-Tracking Data and Its Processing

Gazing behavior was recorded using a Tobii Eye-Tracking System EyeX (Tobii AB, Stockholm, Sweden). The Tobii EyeX Controller uses near-infrared light to track the eye movements, the fixations, and gaze point of a user. The device provides data at a time resolution of 60 Hz and can capture the human gaze pointing at a screen point up to a dimension of the screen of 27′′. This eye-tracking system was set on the desk in front of the subject, between the subject and the screen.

Pre-processing of eye-tracking data recorded by the Tobii EyeX Controller was implemented into the RadarVision software. Fixations are detected when the captured gaze points are located within an area of around 0.2% of the screen for at least 20 ms. For each fixation, the software automatically recorded the relative *x* and *y* on-screen position, the type of object (aircraft/route point) looked at, and its ID. Additionally, timestamps of start and end of each fixation were recorded. All data were written to the database in a separate table. Object type and ID allowed for a definite assignment to all other data of the respective object such as its absolute position in airspace at the time of each fixation. It has to be noted that because the database’s timeticks were based on seconds, it was not possible to further distinguish fixation durations below 1 s. However, it was possible to record multiple fixations occurring within 1 s and save them to the database without loss of information.

#### Additional Measures

In addition to the objective measurements gathered from EEG and eye-tracking, subjective measures of mind wandering and workload were assessed using post-trial questionnaires after each experimental scenario (i.e., BASELINE and SOLUTION). The former was assessed using an adapted version of the DSSQ, the latter using the NASA-TLX. Both questionnaires were prepared as electronic online questionnaires and presented on a tablet.

In particular, the DSSQ was used as a measurement of mind wandering episodes (Cavalcanti and Azevedo, 2013). More precisely, it contains a “Thinking Content” component that can be interpreted as an indicator of mind wandering experienced while performing a task (Smallwood et al., 2009). This component further consists of two sub-scales: “Task-Related Interference” and “Task-Unrelated-Thought.” Both can be used as an indicator of mind wandering episodes. In addition to those two sub-scales, a scale of five items regarding specific “Task-Related Thoughts” was inserted within the DSSQ questionnaire.

The DSSQ items required subjects to rate how often they thought about different things during the last scenario they had completed. All items were given in a conjoint table starting with the phrase: “During the last scenario, I thought about … [*item*]” (e.g., item = *the current traffic situation*). Frequency of respective thoughts was rated on a five-point Likert scale from 1 (Never) to 5 (Very often). Items were randomly arranged between subjects and scenarios to control for sequence effects.

The NASA-TLX questionnaire was used to evaluate overall mental workload along six dimensions, specifically Mental Demand, Physical Demand, Temporal Demand, Effort, Frustration, and Performance (Hart and Staveland, 1988). First, participants were asked to rate the extent of each dimension during the last scenario they had completed. Ratings were given using a horizontal line, ranging from “Low” to “High” on a scale from 0 to 20. Then, all paired combinations of the six dimensions (15 comparisons) were presented to the subjects: for each pair, subjects should decide which of them they deemed more important to how demanding the last scenario was. Those pairwise comparisons were later used to weigh the ratings of each dimension and calculate an overall workload score.

### Performed Analyses

#### EEG-Based Vigilance Scores

The Vigilance scores computed online on the basis of the Controller’s brain activity (i.e., EEG) were averaged over both the experimental scenarios (i.e., BASELINE and SOLUTION) and compared through a two-sided signed Wilcoxon test. Also, on the basis of such indexes, the time length (as percentage of the whole scenario) of the experimental segments classified as “Low Vigilance” condition was calculated for each ATCo. Also, these data were compared through a two-sided signed Wilcoxon test. In general, non-parametric tests, as the Wilcoxon one, have been used each time the data distribution was not Gaussian.

#### Eye-Tracking Data

Eye gaze behavior served as an indicator of vigilance and attention in both scenarios. This included the eye fixations per second and the Time-to-First-Fixation (TTFF) for each aircraft presented during a scenario. The former was used as an indicator of general activity and therefore vigilance. The latter was used as an indirect indicator of vigilance, since a more attentive controller was hypothesized to earlier fixate on newly introduced aircraft than a less attentive one. Scientific literature support this assumption, since the negative correlation between vigilance and reaction times is largely demonstrated (Buck, 1966; Dongen et al., 2005). As Gaussian distribution was not given for fixation data, Wilcoxon tests were performed to analyze differences in fixations per second between scenarios and vigilance levels. A two-way analysis of variance (ANOVA) was performed to investigate eventual effects on the TTFF data, *between* variables of scenarios (BASELINE vs. SOLUTION) and *within* vigilance levels (Low vs. High).

#### Additional Measures

Scores obtained for each subject through both the questionnaires (i.e., NASA-TLX and DSSQ) have been compared through two-tailed paired samples Student’s *t*-test.

## Results

In the following, the results obtained from the data gathered through the methods described above are reported. As previously introduced, the VAC aimed (1) to measure the current vigilance level and the attention focus of the human operator with the aim of detecting or anticipating typical OOTL performance issues, and (2) to adapt automation in case of vigilance decrement with the aim of compensating it.

### EEG-Based Vigilance Scores

**FIGURE 4:**
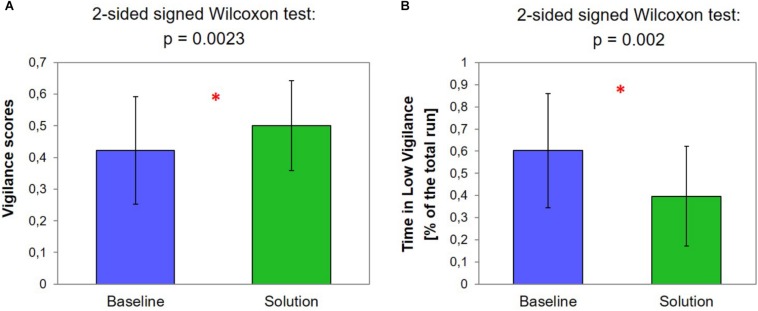
**(A)** Average EEG-based vigilance scores and the distributions measured during the two scenarios. The red asterisk indicates the significant result of the statistical analysis. **(B)** Time percentage of the scenarios classified as “Low Vigilance” on the basis of EEG-based vigilance scores. The red asterisk indicates the significant result of the statistical analysis.

In Figure 4A, the average Vigilance score and the distributions measured during the two scenarios are shown (Baseline: 0.42 ± 0.17; Solution: 0.51 ± 0.14). The two-sided signed Wilcoxon test highlighted a significant increase (*p* = 0.0023) of the overall vigilance scores during the SOLUTION scenario, i.e., it kept the Controller more vigilant.

In Figure 4B, the time percentage of the scenarios classified as “Low Vigilance” is shown (Baseline: 0.60 ± 0.26%; Solution: 0.39 ± 0.22%). The two-sided signed Wilcoxon test highlighted a significant decrease (*p* = 0.002) of the time spent by the Controller in a “Low Vigilance” condition during the SOLUTION scenario, i.e., again it was able to keep him more vigilant.

**FIGURE 5:**
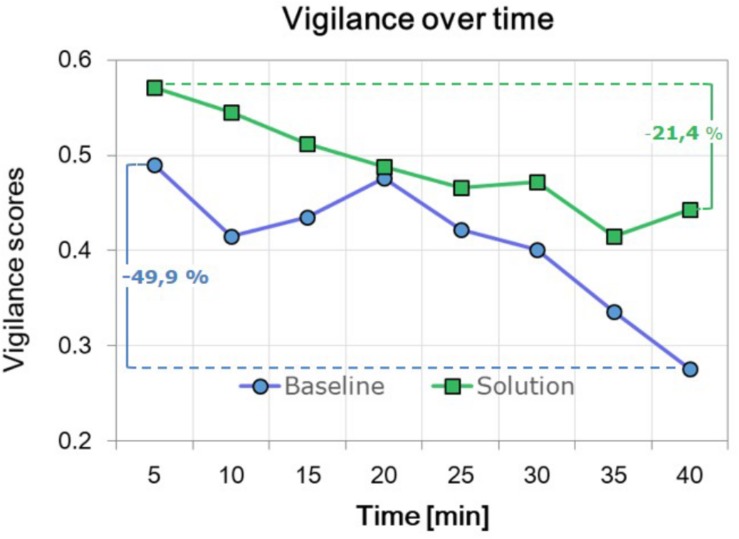
EEG-based vigilance scores evolution along the time (the scenario has been divided in 5-min-long windows to facilitate the representation), averaged between the subjects for both the scenarios. The mean decrease of the vigilance level between the last and the first 5 min, as the percentage with respect to the latter, is also indicated.

Finally, Figure 5 shows the Vigilance scores evolution along the time (the scenario has been divided in 5-min-long windows to facilitate the representation), averaged between the subjects for both the scenarios. It is not possible to perform any statistical analysis, since (i) the subjects number (14) should be at least one magnitude order higher than observations, and (ii) very likely each subject experienced vigilance decreasing in different moments of the task; thus, it is difficult to select two segments to compare. However, the higher decreasing trend of the Vigilance scores for the baseline scenario is evident, as highlighted by the mean decrease (across subjects) of the vigilance level during the last 5 min with respect to the first ones.

### Eye-Tracking Data

**FIGURE 6:**
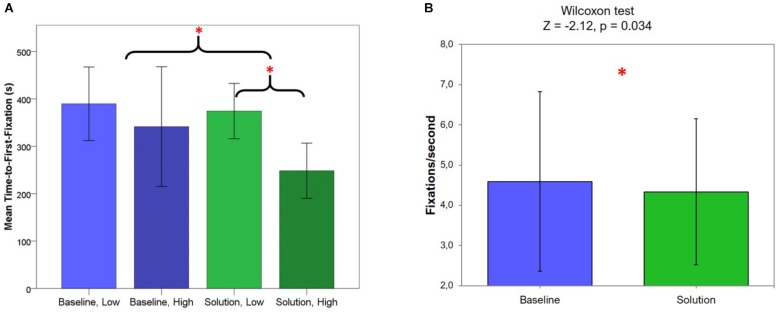
**(A)** Average TTFF and related distributions measured during the two scenarios, and during the sub-segments classified through the EEG data as “Low Vigilance” and “High Vigilance.” The red asterisk indicates the significant result of the statistical analysis. **(B)** Average fixations/second and related distributions measured during the two scenarios. The red asterisk indicates the significant result of the statistical analysis.

A two-way ANOVA was calculated to test differences in TTFF between scenario and vigilance levels for statistical significance (Baseline Low: 5 ± 3 s; Baseline High: 4 ± 1.47 s; Solution Low: 4 ± 1.92 s; Solution High: 4 ± 1.71 s). The results showed a significantly lower TTFF in the whole SOLUTION scenario (*F* = 5.27, *p* = 0.045, η = 0.35) with respect to the BASELINE one. In addition, as depicted in Figure 6A, TTFF during the SOLUTION scenario was also significantly (*p* < 0.05) lower during High Vigilance condition with respect to the Low Vigilance condition.

In addition, Wilcoxon test performed on the fixations/second indicator (Figure 6B) revealed significantly less fixations during the SOLUTION scenario (4.3 ± 1.8 fixations/s) compared to the BASELINE one (4.6 ± 2.2 fixations/s; *p* = 0.034).

### Additional Measures

**FIGURE 7:**
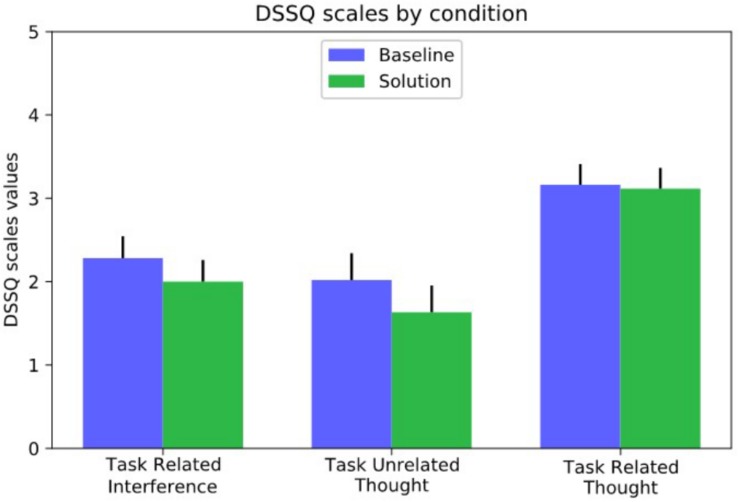
Average scores and related distributions of each item of the DSSQ with respect to the two scenarios.

Finally, the analysis of the self-assessed measures showed the subjective perception of the Controller. In terms of DSSQ results, paired *t*-tests were calculated to test differences in the mind wandering dimensions between BASELINE and SOLUTION scenarios. No significant differences were found for any of the scales (0.59 < *t* < 1.91; 0.098 < *p* < 0.576). However, we observe a decrease in non-relevant thought (task-related interference and task unrelated-thought) in the SOLUTION scenario, whereas task-related thoughts seem equivalent between the two conditions (Figure 7).

**FIGURE 8:**
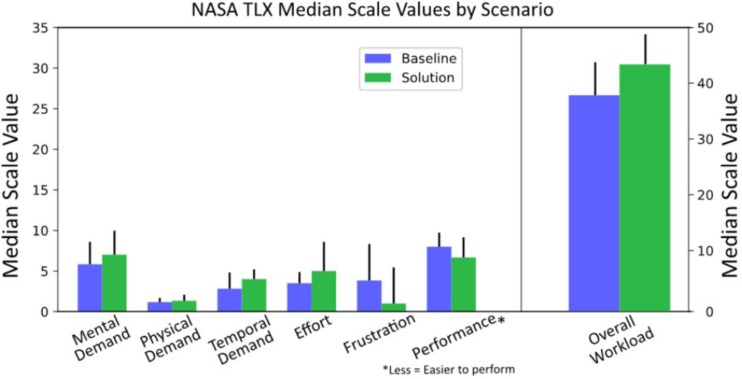
Average scores and related distributions of each item of the NASA-TLX with respect to the two scenarios.

In terms of NASA-TLX, Wilcoxon tests were calculated to test differences among its dimensions between BASELINE and SOLUTION scenario. None of the tests revealed significant differences in the different workload scales. However, as observed for DSSQ results, it has to be noted that all differences, although not statistically significant, showed similar trends (Figure 8): Demands, Effort, and Overall Workload had higher absolute mean values in the SOLUTION scenario (Overall Workload, Baseline: 38 ± 7; Solution: 43 ± 6) while Frustration and Performance had lower (= less dissatisfaction) mean values in the SOLUTION scenario.

## Discussion

The aviation domain is facing a constant growth in terms of passengers and flights, as well as new types of vehicles (i.e., UAVs) bustling about the skies; therefore, the air traffic management has to handle this increasing complexity of air traffic dynamics. Automation is considered the key to support ATCos during the future operative activities; however, the benefits of extreme automation levels are still debated in literature (Parasuraman et al., 2000; Aricò et al., 2017b). The concept of AA, i.e., a system able to allocate operative tasks to the machine or to the human operator depending on the situation and the operator status, has been pointed out as the final solution to mitigate those human factor issues related to high automation, generally summarized with the term “OOTL phenomenon” (Endsley, 1995; Parasuraman et al., 1996).

This study aimed at describing and validating the so-called VAC, a system based on EEG and eye-tracking techniques, aimed to assess in real time the vigilance level of an ATCo dealing with a highly automated interface and to use this measure to adapt the LOA of the interface itself. The interface has been designed according to what is expected in the next decades, i.e., with the highest LOA. However, the interface automation level can be lowered through proper external triggers, in this case provided by the VAC. The 14 ATCos involved in the study performed two high realistic scenarios, one with the VAC disabled (BASELINE scenario, i.e., the automation level was kept fixed at the maximum level during the whole scenario) and one with the VAC system enabled (SOLUTION scenario, i.e., AA).

The results of the EEG data analysis appear to support the theory about benefits of adapting the LOA of the system. In fact, the overall EEG-based Vigilance scores during the SOLUTION scenario were significantly higher (*p* = 0.0023) than during the BASELINE scenario (Figure 4A). This effect is highlighted by the group trends of the EEG-based vigilance scores across the whole scenarios (Figure 5). A monotonous decrease in vigilance during the BASELINE scenario is evident. Consequently, since automation was constantly kept at a high level at all times, vigilance continued to decrease over time. Although a recovery phase was indicated in the middle of the scenario, vigilance continued to decrease after that, and further below the vigilance level as it was prior to the recovery phase. However, since each subject probably experienced vigilance decreasing in different moments of the scenario, fluctuations along the time are less important than the overall trend. Actually, vigilance was also found to decrease during the SOLUTION scenario, when automation level was dynamically adapted by the VAC. This further supports the general hypothesis on the negative impact of high automation levels on controller vigilance. However, while the trend of Vigilance scores during the BASELINE scenario is monotonously decreasing, during the SOLUTION scenario, the decreasing trend seems to be moderated and results in a plateau, most likely because of the effect of AA. The results confirmed this interpretation, since while during the BASELINE scenario the ATCos showed a Vigilance decrease of 49.9%, during the SOLUTION scenario, such a decrease has been quantified in 21.4% with respect to the initial level of Vigilance (Figure 5). Both scenarios were virtually equal when automation was high. Therefore, it can be assumed that differences in controller vigilance did not stem from systematic bias due to difference in traffic, but actually resulted from lack of active involvement. The results in terms of time percentage classified as “Low vigilance” supported these conclusions: in fact, the time spent by the ATCos in a “Low vigilance” condition was significantly higher (*p* = 0.002) during the BASELINE than during the SOLUTION scenario (Figure 4B). Reasonably, this is a consequence of the fact that, while during BASELINE in case of “Low vigilance” detection nothing happened, during SOLUTION, the system effectively reacted to a “Low vigilance” state occurrence reducing its automation level, i.e., allocating the control of operative tasks to the ATCo, thus involving him again *in-the-loop*. A countercheck of this conclusion would rely on recording a scenario closed to the SOLUTION one, but with AA randomly triggered. In this configuration, controllers’ vigilance decrease would be equally mitigated, but in a less effective way, since the automation level adaptation will not be timely, i.e., synchronous with the ATCo’s necessities. This experiment was not performed within the present study, but it is a factor that should be taken into account in future studies.

The results of the eye-tracking data analysis supported the highlights coming from EEG data analysis, i.e., a vigilance decrease induced by high LOAs (BASELINE scenario) and mitigated by the adoption of AA triggered by the VAC (SOLUTION scenario). In particular, TTFF, i.e., the difference in aircraft onset time and the time at which it was first fixated by controllers, were found to be significantly lower (*p* = 0.045, Figure 6A) during the SOLUTION scenario. Therefore, incoming aircraft were recognized earlier and controllers more carefully processed information during fixations. This is coherent with previous literature (Bang and Wojdynski, 2016) and consistent with the higher average level of vigilance in the SOLUTION scenario as shown by the EEG results. In addition, during the BASELINE scenario, no differences arose between Low and High Vigilance segments, since actually nothing changed within the scenario. On the contrary, during the SOLUTION scenario, the TTFF was significantly lower (*p* < 0.05, Figure 6A) during the High Vigilance condition with respect to the Low Vigilance condition, confirming the positive impact of AA solutions. Regarding the results in terms of eye fixations, revealing a significantly higher number of fixations during the BASELINE scenario (*p* = 0.03, Figure 6B), the higher number of fixations per second could be seen as a higher level of monitoring activity. However, it does not necessarily mean that controllers actually carefully process what they look at. Not surprisingly, it has been demonstrated that lower durations of single fixations could in fact represent undirected gazing behavior without any conscious perception (Van Orden et al., 2000). In fact, a lower mean duration would naturally result in more fixations per second and vice versa. Thus, assuming that higher mean duration of fixations (and therefore fewer fixations per second) indicated a more conscious processing of perceived information, it meant that a vigilance activity was higher during the SOLUTION scenario.

Therefore, the eye-tracking data show that the neurophysiological reactions to lack of involvement also result in observable changes in controller behaviors.

In addition to the objective measures, questionnaires were used to get a subjective insight into how controllers perceived their thinking in terms of workload and mind wandering.

In terms of the mind wandering sub-scales used in the DSSQ (Figure 7), during the SOLUTION scenario, controllers reported to have experienced less task-related interference and less task-unrelated thoughts compared to the BASELINE scenario. Therefore, controllers were less likely to be distracted by other matters beside their task. Regarding task-related thoughts, mean values of BASELINE and SOLUTION scenario were virtually equal. This indicates that controllers equally thought about things related to their task no matter how actively they were involved in it.

Analyses of the NASA-TLX data assessed through the questionnaire (Figure 8) revealed very intriguing results. Median values of mental, physical, and temporal demand were higher in the SOLUTION scenario. This indicates that during the SOLUTION scenario, controllers perceived the task to require them to think more, act more, and do so in a more time-critical manner. In total, controllers felt like there was “more to do” during the SOLUTION scenario. Concerning the remaining sub-scales of the NASA-TLX (Effort, Frustration, and Performance), the results were also in accordance with these preliminary conclusions. Usually, it is expected that increasing demand results in more effort, more frustration, and less performance. However, the VAC concept aimed to result in more effort as it puts the human operator back in the loop. It also aims to lower frustration stemming from the lack of involvement and increase performance. Actually, ATCos rated the task to be less frustrating during the SOLUTION scenario. Finally, it was stated that it was easier for them to achieve good performance during the SOLUTION scenario. It is likely that those results stem from the higher degree of active task involvement during the SOLUTION scenario as their role was shifted from a mere passive monitor to an actively involved controller. Results also showed that overall workload was higher during the SOLUTION scenario, although the increase was very small and not significant. This result is fully aligned with the theory of the inverted U-shape relationship between human performance and workload: a workload increase, until a certain threshold, can be productive for human performance, since it results in a higher engagement (Westman and Eden, 1996; Pop et al., 2012).

In conclusion, the NASA-TLX results show that controllers perceived the SOLUTION scenario to be more demanding, less frustrating, and easier to achieve good performance in. However, none of the differences achieve statistical significance due to the very limited power from the small sample and the related poor sensitivity of subjective measures (Aricò et al., 2016b; Scerbo, 1996).

Unfortunately, it was impossible to employ any measure about ATCos’ performance, since if automation takes total control of the system, no manual actions are required to them; therefore, it is impossible to have a measure of their performance. However, this is actually a problem related to futuristic systems; i.e., it would be more and more difficult, even impossible, to have a feedback about user’s performance; therefore, alternative techniques able to produce information about the user’s state will become popular.

Because of that, further research involving, first of all, a larger experimental sample is needed. The involvement of professional figures and facilities is undoubtedly costly and time demanding, but the results of this preliminary research would encourage further investigation about this issue, in order to obtain stronger evidences and large-scale validation of the proposed solutions. Additionally, this study only compares two very different conditions, i.e., a fully automatic (Level 2 of automation) and a highly manual one (Level 0 of automation), to enhance the difference in terms of expected mental behaviors. Future research would also include different LOAs and maybe intermediate ways of interaction, in order to have a comprehensive evaluation of the issue.

However, despite the demanding constraints due to the realistic settings and the professional figures and facilities involved within the experiments, the study succeeded (i) to elicit a different mental and overt behavior of the participants depending on the LOA they were interacting with, and (ii) to provide measures of such differences. Therefore, the reader has to expect the work to be an applied example of the *Macrocognition* theory, i.e., a descriptive level of cognition performed in natural instead of artificial (laboratory) environments (Klein et al., 2003). In particular, the present study pointed out very intriguing results, especially if the highly realistic experimental environment is considered: (i) it has demonstrated how prolonged high LOAs induce vigilance decreasing on humans; (ii) it has demonstrated how AA-based systems are able to counteract such vigilance decrease, thus mitigating the risk of OOTL phenomena; (iii) neurophysiological measures, in particular EEG, have been demonstrated to be a reliable and effective tool to trigger such AA-based systems. These results are even more interesting if the recent progress in the sensor industry in developing minimally invasive EEG devices and techniques is considered (Borghini et al., 2019; Di Flumeri et al., 2019), opening the doors to new frontiers of augmented human–machine interaction.

## Conclusion

Nowadays, the role of operators in general, and in this specific case ATCos, is shifting from an *active* operator handling different tools and functions to a mere *supervisor* of highly automated interfaces. The latter induces a series of human factor issues included in the concept of the OOTL phenomenon. On one hand, this work confirmed the expected negative impact of fully automated interfaces, which induced a constant decreasing of operators’ vigilance, reflected by a higher frustration and unsatisfaction as well as a tendency to make task-unrelated thoughts while operating. On the other hand, this work highlighted (i) the properness and reliability of the EEG technique as an information channel to monitor online the vigilance level of the operator and to trigger the control logic of AA-based systems, and (ii) the effectiveness of AA-based systems in counteracting the vigilance decrease induced by highly automated systems. The controllers themselves were the best-performing, were more engaged in the task, have less task-unrelated thoughts, and showed a higher reacting gaze behavior. Despite the limitations of the present study, intended as preliminary research, the intriguing results encourage further research in this field.

The application of this kind of technology, i.e., passive BCIs, would enhance the cooperation among humans and machines, increasing the overall system performance and resulting in higher safety standards.

## Data Availability

The datasets generated for this study are available on request to the corresponding author.

## Ethics Statement

The experiment was conducted following the principles outlined in the Declaration of Helsinki of 1975, as revised in 2000. Informed consent and authorization to use the video graphical material were obtained from each subject on paper, after the explanation of the study. The protocol was approved by the Ethical Committee of University of Bologna, since the experiments took place at the Virtual Reality Lab of the University of Bologna (in Forlì, Italy).

## Author Contributions

GD, GB, FD, BB, JK, and OO contributed to conception and design of the study. OO and JK developed the ATM simulator. FD and FB supervised the experiments organization. FD, SB, and SP took care of experiments execution. GD and PA performed the neurophysiological measures. GD, GB, and PA analyzed the neurophysiological measures. JK and OO provided support for the ATM simulator and analyzed eye tracker data. GD wrote the first draft of the manuscript. FB, PA, GB, SP, BB, and OO wrote sections of the manuscript. All authors contributed to manuscript revision, read and approved the submitted version.

## Conflict of Interest Statement

GD, PA, GB, and FB were employed by the company BrainSigns srl. The remaining authors declare that the research was conducted in the absence of any commercial or financial relationships that could be construed as a potential conflict of interest.
